# Assessing agreement with a single-center expert consensus: artificial intelligence-assisted teleultrasound for thyroid nodules categorized as C-TIRADS 4A or higher

**DOI:** 10.3389/fendo.2026.1646240

**Published:** 2026-03-25

**Authors:** Cuo Yi, Xue Li, Ting Peng, Lin Li, Rong Chen, Xi Yang

**Affiliations:** 1 Department of Ultrasound, Shapingba Hospital affiliated to Chongqing University (Shapingba District People’s Hospital of Chongqing), Chongqing, China; 2 Tuzhu Community Health Service Center, Chongqing, China; 3 Shapingba Community Health Service Center, Chongqing, China

**Keywords:** artificial intelligence, telemedicine, ultrasonography, thyroid nodule, community health centers

## Abstract

**Objective:**

To evaluate the diagnostic agreement between artificial intelligence (AI)-assisted teleultrasound and expert consensus in thyroid nodules classified as Chinese Thyroid Imaging Reporting and Data System (C-TIRADS) 4A or higher.

**Methods:**

This retrospective study enrolled 419 patients with 587 thyroid nodules examined at the Chongqing Shapingba District Community Health Service Center between January 2024 and June 2024. Among these, 80 thyroid nodules diagnosed as C-TIRADS 4A or higher (excluding category 5) by community medical institutions or teleultrasound were further analyzed using an AI diagnostic system. The expert consensus of three teleultrasound specialists from a single center served as the reference standard. Diagnostic agreement between the community medical institutions, teleultrasound, and the AI system was compared in this study.

**Results:**

Diagnostic consistency between community medical institutions and teleultrasound was poor (linear weighted kappa = 0.20 [95% confidence interval (CI): −0.04 to 0.44]), whereas good diagnostic consistency was observed between teleultrasound and the AI system (linear weighted kappa = 0.80 [95% CI: 0.67, 0.93]). Receiver operating characteristic (ROC) curve analysis revealed that community medical institutions showed significantly lower diagnostic performance for nodules classified as C-TIRADS 4A or higher (macro-average area under the curve (AUC) = 0.55 [95% CI: 0.45, 0.65]). In contrast, the AI system achieved comparable diagnostic performance to teleultrasound (macro-average AUC = 0.92 [95% CI: 0.81, 0.97]; paired t-test: t = 165.92, *p* < 0.001; bootstrap [95% CI: 0.21, 0.49]). At the ≥C-TIRADS 4A threshold, the AI system yielded a sensitivity of 97.1% [95% CI: 90.2, 99.2] and a specificity of 100.0% [95% CI: 72.2, 100.0]. At the ≥C-TIRADS 4B threshold, sensitivity and specificity were 100.0% [95% CI: 81.6, 100.0] and 90.5% [95% CI: 80.7, 95.6], respectively.

**Conclusion:**

The AI system can improve the diagnostic agreement of community medical institutions in evaluating thyroid nodules classified as C-TIRADS 4A or higher, achieving assessment consistency comparable to expert-level teleultrasound assessments.

## Introduction

Thyroid cancer is among the most common malignancies in the head and neck region (1). Current evidence suggests a trend of overdiagnosis and overtreatment of thyroid cancer, leading to unnecessary biopsies and surgeries (2). To mitigate this issue, clinical guidelines recommend active surveillance for small, low-risk thyroid cancers rather than immediate surgical interventions (3–6). Accurate risk evaluation and diagnosis are therefore essential for the appropriate implementation of active surveillance, particularly for thyroid nodules categorized as C-TIRADS 4A or higher. These nodules typically require ultrasound follow-up or biopsy and represent a key decision point in clinical management (7, 8).

Ultrasound remains the primary imaging modality for thyroid cancer evaluation (9, 10). The 2015 American Thyroid Association (ATA) Management Guidelines for Adult Patients with Thyroid Nodules and Differentiated Thyroid Cancer recommend ultrasonography for all patients with suspected thyroid nodules, nodular goiter, or imaging abnormalities (4). As a non-invasive and highly sensitive technique, ultrasound allows comprehensive assessment of nodule number, location, and features associated with malignancy (11, 12).

In China, primary care institutions represent the frontline of the healthcare system, providing medical services to approximately 50% of the population (13) and playing a central role in the early detection of thyroid cancer. However, these settings face a severe shortage of sonographers (14) due to recruitment challenges and high staff turnover (15). Additionally, the lack of systematic training programs often results in variable diagnostic proficiency and non-standardized protocols, which contribute to the misdiagnosis of thyroid nodules.

Teleultrasound has been validated as a reliable approach to enhance diagnostic accuracy of junior practitioners (16–18). Since 2021, our teleultrasound platform has conducted quality control on over 370,000 ultrasound reports with daily report reviews exceeding 1,000 cases during peak periods. Such heavy workload increases the risks of diagnostic errors and oversight. Therefore, innovative solutions are urgently needed to improve the efficiency and accuracy of thyroid ultrasound quality control.

AI, particularly deep learning systems, has emerged as a transformative tool for ultrasound image analysis. Substantial evidence confirms that AI can objectively quantify thyroid nodule features and facilitate malignancy differentiation (19–21). Our institution employs the Yi Zhun 4.0 AI system interoperable with the teleultrasound platform. Pretrained on large-scale data, this system enables real-time nodule localization, feature extraction, and C-TIRADS classification predictions.

This study introduces several novel aspects compared to prior literature. While previous studies have predominantly focused on AI-based analysis of ultrasound images acquired directly from diagnostic devices, our study obtained images transmitted via a teleultrasound platform. However, domain shift may impair AI interpretability due to image quality degradation (22, 23), including lossy compression, resolution changes, or artifacts during transmission (24). Such degradation can occur even under nominally “lossless” Digital Imaging and Communications in Medicine (DICOM) (1.2.840.10008.1.2.4.70) compression, which may alter the image metadata or dynamic range—an underexplored yet critical issue. Furthermore, whereas prior work has emphasized AI diagnostic accuracy, our investigation specifically evaluates its agreement with expert consensus in the interpretation of transmitted images, providing a more clinically relevant validation benchmark. To our knowledge, our work represents one of the first efforts to integrate AI assistance directly into the teleultrasound workflow for thyroid nodule assessment, thereby addressing the persistent challenges of substantial workload and quality control in primary care settings.

Therefore, this study aims to test the hypothesis that AI-assisted teleultrasound achieves non-inferior diagnostic agreement with expert consensus for thyroid nodules classified as C-TIRADS 4A or higher.

## Materials and methods

**Figure 1:**
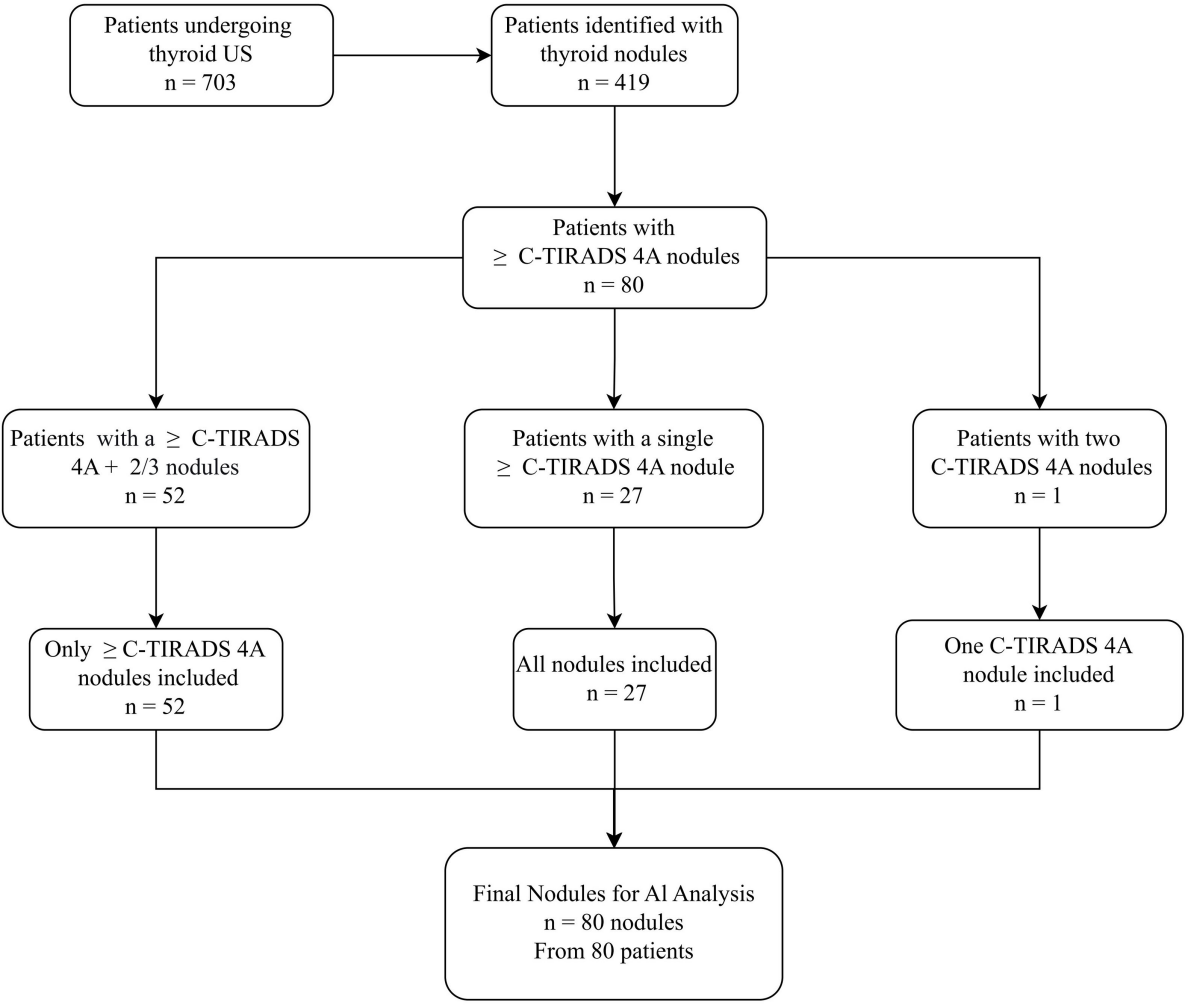
Flowchart of patients and thyroid nodule selection.

This retrospective study collected thyroid ultrasound images uploaded to a teleultrasound system from patients who underwent thyroid ultrasound examinations at the Chongqing Shapingba District Community Health Service Center between January 2024 and June 2024. Patients diagnosed with thyroid nodules were included, and data from 19 of the 22 participating community medical institutions were ultimately analyzed. The final AI analysis comprised 80 thyroid nodules from 80 patients, all classified as C-TIRADS 4A or higher. The overall workflow is depicted in **Figure 1**. This study was approved by the Ethics Committee of People’s Hospital of Shapingba District, Chongqing (Approval No. KY202341). The requirement for written informed consent was waived by the ethics committee.

### AI system

The Yi Zhun AI model was trained on freehand ultrasound videos and static images (25). It is built upon a composite deep-learning architecture, comprising separate detection (Faster Region-Based Convolutional Neural Network: Faster R-CNN) and classification (Inflated 3D ConvNet: I3D) networks. Predictions are generated by synthesizing information from multiple lesion video clips. To determine C-TIRADS categories, the AI system employs a multi-task framework that identifies key lesion characteristics—such as composition, echogenicity, margins, and other features specified in the C-TIRADS guidelines—and subsequently translates these features into the corresponding C-TIRADS categories based on clinical guidelines (**Table 1**).

**Table 1:** C-TIRADS based on the counting method.

Nodule	Score	Malignancy rate (%)	C-TIRADS category
Absent	NA	0	1, no nodule
Present	−1	0	2, benign
	0	<2	3, probably benign
	1	2-10	4A, low suspicion
	2	10-50	4B, moderate suspicion
	3-4	50-90	4C, high suspicion
	5	>90	5, highly suggestive of malignancy
	NA	NA	6, biopsy proved malignant

*C-TIRADS, Chinese Thyroid Imaging Reporting and Data System; NA, not available.

The model was trained on a large-scale, multicenter dataset consisting of over 10,000 ultrasound videos collected from more than 10 hospitals. No threshold adjustment or *post-hoc* calibration was applied during validation or testing. C-TIRADS categories were assigned strictly according to predefined rules. Upon completion of training, all model parameters were fixed following clinical evaluations to avoid further adjustments or optimizations during deployment.

### Image acquisition and transmission

**Figure 2:**
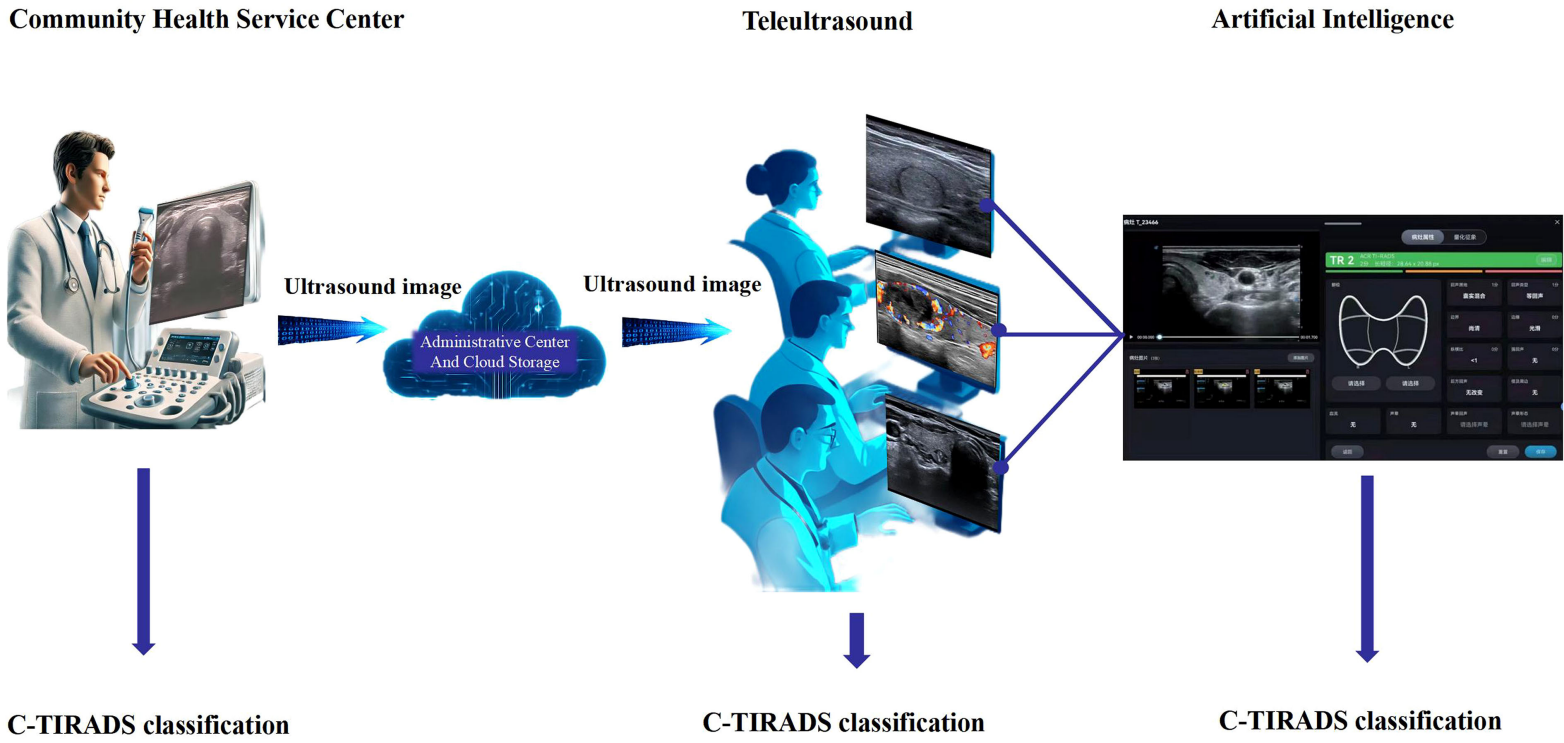
Workflow of ultrasound diagnosis in thyroid nodules.

During the initial deployment of the teleultrasound platform, all staff from participating community institutions received short-term training in ultrasound scanning. Ultrasound examinations were performed using multifunctional color Doppler ultrasound diagnostic devices from multiple vendors, including GE (n = 6), Mindray (n = 7), SonoScape (n = 2), Hitachi (n = 1), Philips (n = 1), Siemens (n = 1), and SIUI (n = 1), all equipped with linear array probes (8–12 MHz). Standardized thyroid ultrasound sections were obtained, with careful documentation of nodule features, particularly characteristics suggestive of malignancy. All images were subsequently uploaded to the teleultrasound platform (**Figure 2**).

The teleultrasound platform employed the lossless compression protocol DICOM 1.2.840.10008.1.2.4.70, which reduced the image size to approximately 30%–40% of the original. Theoretically, the resolution remains unchanged before and after transmission, as lossless compression preserves full image resolution.

Quality control during upload was limited to automated verification of technical criteria, including successful transmission, file format, and data integrity. The system did not perform automated assessment of diagnostic suitability, such as nodule visibility or artifact detection. Consequently, the responsibility for ensuring diagnostic image quality rested with the operational protocols of the community sonographers. To accurately reflect the real-world teleultrasound workflow and evaluate the robustness of the AI model, no preprocessing quality filter or exclusion based on signal-to-noise ratio (SNR) was applied to the images.

### Expert consensus reference and AI analysis

Thyroid nodule ultrasound images uploaded by community medical institutions to the teleultrasound platform were anonymized and presented sequentially according to the order of examination. Three professional ultrasound physicians from our hospital independently evaluated the images in strict accordance with the C-TIRADS classification criteria (26) (detailed in **Table 2**), with each expert blinded to the others’ evaluations. In cases of diagnostic discrepancies, a consensus diagnosis was reached through discussion. This expert consensus was designated as the reference standard for the study, rather than pathological results.

**Table 2:** Baseline characteristics of the study cohort (n=80).

Characteristic	Value/N (%)	
Age, years (mean ± SD, [range])	59.11 ± 12.94 [31, 87]	
Sex, female	66 (82.50%)	
Nodule characteristics (reference: teleultrasound)		
	N (%)	Maximum diameter (mm, mean ± SD)
3	10 (12.50%)	10.15 ± 6.42
4A	53 (66.25%)	10.39 ± 6.10
4B	15 (18.75%)	6.10 ± 2.12
4C	2 (2.50%)	7.43 ± 2.62

Thyroid nodules classified as C-TIRADS 4A or higher—based on either community medical institutions or teleultrasound assessments—were subsequently screened for AI analysis. The Yi Zhun 4.0 AI system was employed to process these images, providing C-TIRADS classification outcomes for these selected nodules. The overall study flowchart is shown in **Figure 1**.

### Statistics

All statistical analyses were performed using R software (version 4.5.0). C-TIRADS classifications for thyroid nodules were treated as ordinal data. Agreement between each diagnostic method—community physicians and the AI system—and the reference standard (teleultrasound expert consensus) was quantified using the linear weighted Kappa statistic with corresponding 95% CI. Kappa values were interpreted as follows: 0–0.20, poor agreement; 0.21–0.40, fair agreement; 0.41–0.60, moderate agreement; 0.61–0.80, good agreement; and 0.81–1.00, excellent agreement.

Using teleultrasound diagnostic results as the reference standard, the diagnostic performance of the AI system and community physicians was evaluated. Sensitivity, specificity, positive predictive value (PPV), and negative predictive value (NPV) were calculated with their 95% CI using the Clopper–Pearson exact method. Performance was assessed at two clinically relevant thresholds (C-TIRADS ≥4A and ≥4B) to reflect distinct clinical decision pathways in thyroid nodule management.

ROC curves were generated for both diagnostic sources with the teleultrasound consensus as the reference. The AUC was employed to evaluate concordance with the expert consensus, with optimal operating points identified. AUCs and their 95% CI were calculated, with differences between community physicians and the AI system compared using DeLong’s test for individual C-TIRADS categories. To compare macro-average AUCs, a paired t-test combined with bootstrap resampling was applied. Statistical significance was defined as *p* < 0.05, and a CI for the estimated statistic that excludes zero indicates a statistically significant difference in bootstrap resampling.

## Results

### General information

A total of 419 patients with 587 thyroid nodules were included in the study. Among these, 80 thyroid nodules classified as C-TIRADS 4A or higher (excluding category 5) based on malignant sonographic features were further analyzed by community medical institutions and the teleultrasound using AI assistance. The baseline characteristics of the study cohort are shown in **Table 2**.

### Diagnostic agreement and performance

The weighted Kappa analysis revealed poor agreement between community medical institutions and teleultrasound (linear weighted kappa = 0.20 [95% CI: −0.04, 0.44]), whereas the diagnostic consistency between teleultrasound and the AI system was good (linear weighted kappa = 0.80 [95% CI: 0.67, 0.93]). The complete cross-tabulation of the 80 thyroid nodules classified as C-TIRADS 4A or higher by the three methods is presented in **Table 3**.

**Table 3:** Agreement of C-TIRADS classifications between different methods and weighted kappa statistics.

(A) Community health service center vs. teleultrasound (reference)					
Teleultrasound	Community health service center				Total
	3	4A	4B	4C	
	n (% of Teleultrasound Row)				n (% of Total)
3	0 (0.0%)	10 (100.0%)	0 (0.0%)	0 (0.0%)	10 (12.5%)
4A	10 (18.9%)	40 (75.5%)	2 (3.8%)	1 (1.9%)	53 (66.3%)
4B	1 (6.7%)	11 (73.3%)	3 (20.0%)	0 (0.0%)	15 (18.8%)
4C	0 (0.0%)	1 (50.0%)	0 (0.0%)	1 (50.0%)	2 (2.5%)
Total	11 (13.8%)	62 (77.5%)	5 (6.3%)	2 (2.5%)	80 (100.0%)

(B) Artificial intelligence vs. teleultrasound (reference)					
Teleultrasound	Artificial intelligence				Total
	3	4A	4B	4C	
	n (% of teleultrasound row)				n (% of total)
3	10 (100.0%)	0 (0.0%)	0 (0.0%)	0 (0.0%)	10 (12.5%)
4A	2 (3.8%)	45 (84.9%)	4 (7.5%)	2 (3.8%)	53 (66.3%)
4B	0 (0.0%)	0 (0.0%)	12 (80.0%)	3 (20.0%)	15 (18.8%)
4C	0 (0.0%)	0 (0.0%)	0 (0.0%)	2 (100.0%)	2 (2.5%)
Total	12 (15.0%)	45 (56.3%)	16 (20.0%)	7 (8.8%)	80 (100.0%)

Linear weighted kappa = 0.20 [95% CI: -0.04, 0.44] (poor agreement). linear weighted kappa = 0.80 [95% CI: 0.67, 0.93] (good agreement).

### Diagnostic performance of AI and community physicians against the teleultrasound reference standard

At the ≥C-TIRADS 4A threshold, the AI system achieved a sensitivity of 97.1% [95% CI: 90.2, 99.2] and a specificity of 100.0% [95% CI: 72.2, 100.0]. In comparison, community physicians demonstrated a sensitivity of 84.3% [95% CI: 74.0, 91.0] and a specificity of 0.0% [95% CI: 0.0, 27.8]. At the ≥C-TIRADS 4B threshold, the AI system achieved a sensitivity of 100.0% [95% CI: 81.6, 100.0] and a specificity of 90.5% [95% CI: 80.7, 95.6]. In contrast, community physicians showed a sensitivity of 25.0% [95% CI: 10.2, 49.5] and a specificity of 93.8% [95% CI: 85.0, 97.5]. The corresponding values for PPV and NPV are detailed in **Table 4**.

**Table 4:** Diagnostic performance of AI and community-based ultrasound for thyroid nodules at different C-TIRADS thresholds (reference: teleultrasound).

Methods	thresholds	Sensitivity (95% CI)	Specificity (95% CI)	PPV (95% CI)	NPV (95% CI)
AI	≥ 4A	97.1% [90.2, 99.2]	100.0% [72.2, 100.0]	100.0% [94.7, 100.0]	83.3% [55.2, 95.3]
Community	≥ 4A	84.3% [74.0, 91.0]	0.0% [0.0, 27.8]	85.5% [75.3, 91.9]	0.0% [0.0, 25.9]
AI	≥ 4B	100.0% [81.6, 100]	90.5% [80.7, 95.6]	73.9% [53.5, 87.5]	100.0% [93.7, 100.0]
Community	≥ 4B	25.0% [10.2, 49.5]	93.8% [85.0, 97.5]	50.0% [21.5, 78.5]	83.3% [73.1, 90.2]

PPV, positive predictive value; NPV, negative predictive value.

### ROC curves of different diagnostic methods for C-TIRADS 4A or higher thyroid nodules

Using teleultrasound C-TIRADS classifications as the reference standard, ROC curves were plotted based on the sensitivity and specificity of different diagnostic methods. The macro-average AUC for the AI system was 0.92 [95% CI: 0.81, 0.97], compared with 0.55 [95% CI: 0.45, 0.65] (paired t-test: t = 165.92, *p <* 0.001; bootstrap [95% CI: 0.21, 0.49]).

The diagnostic performance of C-TIRADS categories (AI system vs. community) was as follows: C-TIRADS 3 (0.99 [95% CI: 0.97, 1.00] vs. 0.42 [95% CI: 0.38, 0.46], Delong z score = 28.01, *p <* 0.001), C-TIRADS 4A (0.93 [95% CI: 0.88, 0.97] vs. 0.47 [95% CI: 0.38, 0.56], Delong z score = 9.27, *p* < 0.001), C-TIRADS 4B (0.87 [95% CI: 0.76, 0.98] vs. 0.58 [95% CI: 0.48, 0.69], Delong z score = 2.64, *p* = 0.008), and C-TIRADS 4C (0.97 [95% CI: 0.94, 1.00] vs.0.74 [95% CI: 0.25, 1.00], Delong z score = 0.90, *p* = 0.370).

**Figure 3:**
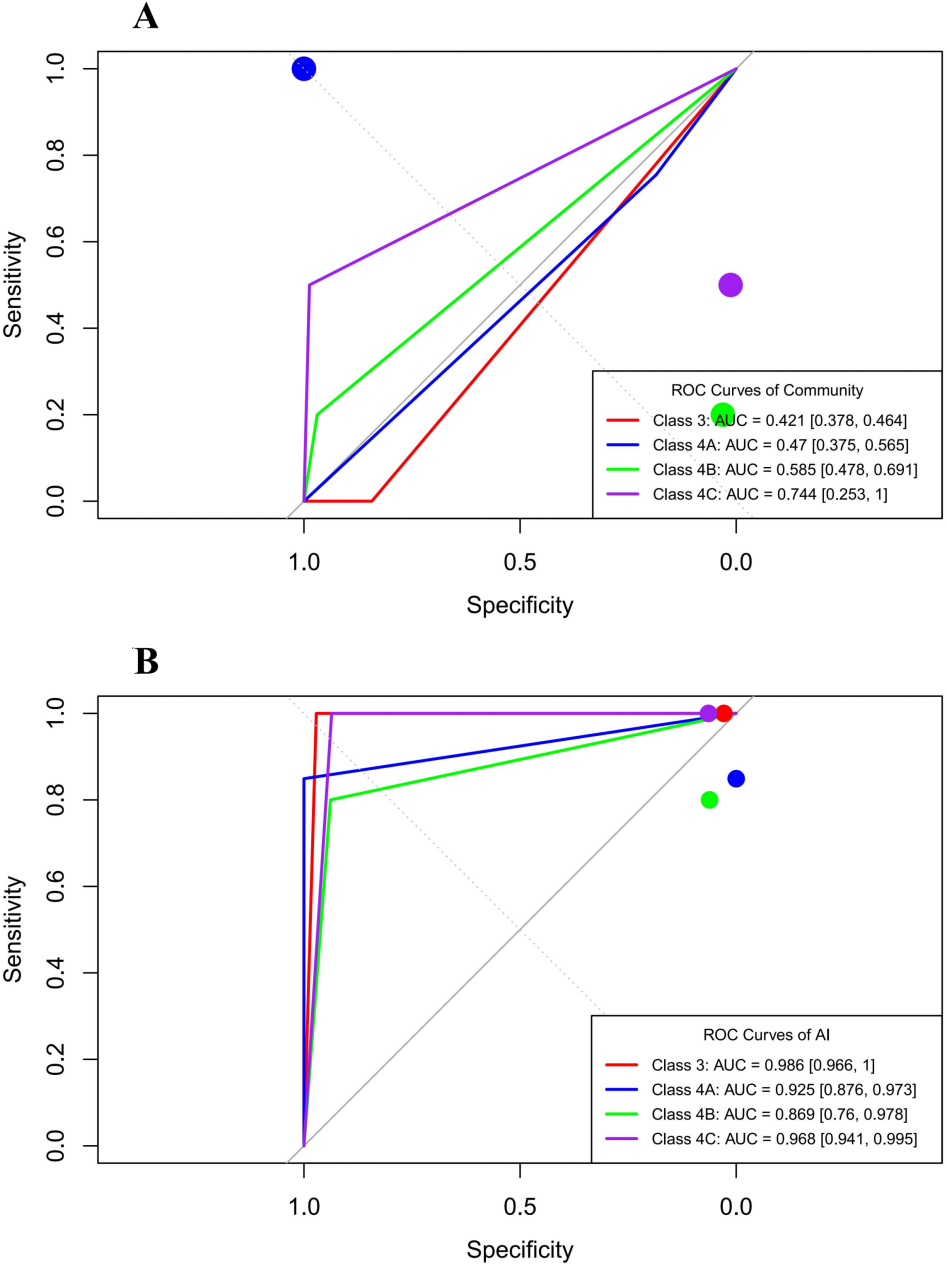
ROC curves for multiclass C-TIRADS classification (reference: teleultrasound). **(A)** Performance of the community health service center. **(B)** Performance of the artificial intelligence system.

At an operating threshold of 0.50, the following results were observed (**Figure 3**). For C-TIRADS 3, the AI system achieved a true positive rate (TPR) of 1.00 and a false positive rate (FPR) of 0.03, whereas the community institutions exhibited TPR and FPR both at 1.00 (threshold = −Inf). For C-TIRADS 4A, the AI system yielded a TPR of 0.85 and an FPR of 0.00, compared with the community TPR and FPR both at 1.00 (threshold = −Inf). For C-TIRADS 4B, the AI system showed a TPR of 0.80 and an FPR of 0.06, whereas the community showed a TPR of 0.20 and an FPR of 0.03. For C-TIRADS 4C, the AI system demonstrated a TPR of 1.00 and an FPR of 0.06, compared with the community with a TPR of 0.5 and an FPR of 0.01.

## Discussion

This study analyzed the ultrasound images of 587 thyroid nodules from 419 patients, among which 80 thyroid nodules classified as C-TIRADS 4A or higher—diagnosed by either community medical institutions or teleultrasound—were included for AI-based evaluation. We conducted an innovative assessment of the diagnostic agreement between AI-assisted teleultrasound and expert consensus for the interpretation of these higher-risk nodules. The study found good consistency between the AI system and teleultrasound (linear weighted kappa = 0.80 [95% CI: 0.67, 0.93]), with diagnostic performance comparable with that of teleultrasound (macro-average AUC = 0.92 [95% CI: 0.81, 0.97]). In contrast, the AI system significantly outperformed community medical institutions, which exhibited poor agreement (linear weighted kappa = 0.20 [95% CI: −0.04, 0.44]) and lower diagnostic accuracy (macro-average AUC = 0.55 [95% CI: 0.45, 0.65], paired t-test: t = 165.92, *p* < 0.001; bootstrap [95% CI: 0.21, 0.49]).

### Comparison with previous studies

Multiple studies have demonstrated that AI can enhance diagnostic performance in thyroid cancer detection (27–30), especially when analyzing high-quality ultrasound images acquired directly from diagnostic devices. However, our study utilized an AI device to directly interpret thyroid nodule ultrasound images uploaded via a teleultrasound platform, which are subject to potential quality degradation during transmission. We further extended prior evidence by confirming AI performance on images transmitted through telemedicine. Moreover, our study focuses on diagnostic agreement with expert consensus rather than diagnostic accuracy against pathology, providing a more clinically relevant benchmark for AI integration into teleultrasound workflows. This shift toward evaluating AI in clinically relevant, real-world telemedicine settings parallels recent developments, such as the implementation of high-accuracy AI models for monkeypox detection in telemedicine platforms (31), and aligns with the broader trend of validating AI as practical diagnostic aids and clinical decision-support tools within real-world clinical workflows (32, 33).

### Diagnostic performance and clinical utility

The AI system demonstrated exceptional sensitivity at clinically critical thresholds, achieving 97.1% [95% CI: 90.2, 99.2] for C-TIRADS ≥4A and 100.0% [95% CI: 81.6, 100.0] for ≥4B, thereby minimizing the risk of missing potentially malignant nodules. Specificity was 100.0% [95% CI: 72.2, 100.0] for ≥4A and 90.5% [95% CI: 80.7, 95.6] for ≥4B. At the ≥4A threshold, the PPV and NPV were 100.0% and 83.3%, respectively; at ≥4B, the PPV and NPV were 73.9% and 100.0%, respectively. Notably, the high NPV at the ≥4B threshold underscores the AI system’s clinical utility in reliably ruling out malignancy, potentially reducing unnecessary clinical interventions.

To evaluate the potential impact of an AI-assisted strategy in a real-world clinical context, we simulated a scenario where nodules classified as ≥C-TIRADS 4B were referred for biopsy, assuming perfect adherence to the referral recommendation. Using expert consensus as the reference standard, the necessary biopsy rate was 15.0% (12/80). If management had been based on community sonographers’ interpretations, the biopsy rate would have been 7.5% (6/80), with a referral rate of 50.0% (6/12), highlighting a significant risk of delayed diagnosis. In contrast, the AI system would lead to a biopsy rate of 25.0% (20/80). Importantly, the AI system could completely eliminate missed referrals (0.0%) but would introduce a higher over-referral rate of 40.0% (8/20). Although the number of biopsies increases with AI assistance, it ensures that all suspicious nodules are identified, offering a safer triage strategy in primary care settings with limited expertise. These findings, however, are based on retrospective data, and the precise clinical benefits should be validated in future prospective studies.

At the defined operating points, the AI system demonstrated excellent performance across all C-TIRADS categories. Specifically, it achieved a TPR of 1.00 and an FPR of 0.03 for C-TIRADS 3; a TPR of 0.85 and an FPR of 0.00 for C-TIRADS 4A; a TPR of 0.80 with an FPR of 0.06 for C-TIRADS 4B; and a TPR of 1.00 with an FPR of 0.06 for C-TIRADS 4C. These operating characteristics underscore the AI system’s ability to maintain high sensitivity while minimizing false positives, supporting its role as a reliable tool not only for ultrasound-based risk stratification but also for optimizing the subsequent clinical pathways, which often involve cytological evaluation.

### Integration with cytological risk stratification and clinical implications

The reliable triage of C-TIRADS 4A or higher thyroid nodules by AI must be interpreted within the broader clinical workflow, which typically proceeds to fine-needle aspiration (FNA) and cytological evaluation using The Bethesda System for Reporting Thyroid Cytopathology (TBSRTC). A persistent challenge in thyroidology is the management of indeterminate cytology. The reported risk of malignancy (ROM) for indeterminate categories has evolved over time, particularly between the first (2009) and second (2017) editions of TBSRTC, primarily reflecting a refined understanding of the disease spectrum, including the introduction of the new classification of non-invasive follicular thyroid neoplasm with papillary-like nuclear features (NIFTP) (34). For instance, the ROM for the indeterminate category AUS/FLUS (category III) increased from 5%–15% to 10%–30%, whereas the ROM for “suspicious for malignancy” (category V) was adjusted from 60%–75% to 50%–75% (34, 35). This evolution underscores both the importance of precise risk stratification and the dynamic nature of cytopathological diagnosis. Within this framework, the subdivision of TBSRTC category III (AUS/FLUS) represents a crucial issue, as it is a heterogeneous entity with ROM that may vary significantly based on specific cytomorphological features (35). Furthermore, the broader definition of “indeterminate cytology” often encompasses TBSRTC Categories III, IV, and V, as noted in major guidelines and commentaries, which carries implications for ROM and management strategies (34, 36).

Our AI-assisted teleultrasound paradigm directly addresses upstream challenges in the thyroid diagnostic cascade. By providing a highly consistent, expert-level ultrasound risk assessment at the community level, our system can help better identify C-TIRADS 4A or higher nodules that warrant FNA. This preselection has the potential to reduce the number of nodules yielding indeterminate cytology results (Bethesda III–V), thereby streamlining patient management and mitigating diagnostic uncertainty. Such an approach aligns with the ongoing efforts to improve pre-cytology triage. The subdivision of “intermediate suspicion” categories in ultrasound systems, such as the 2021 Korean Thyroid Imaging Reporting and Data System (K-TIRADS), reflects a parallel effort to refine risk stratification (35), and our AI-based approach contributes to this endeavor. Moreover, the demonstrated robustness of our AI system is a prerequisite for reliable integration into workflows where FNA quality is paramount; ongoing debate regarding optimal needle gauge (e.g., 22-, 23-, 25-, or even 27-gauge) to minimize non-diagnostic rates further highlights the critical need for precise and reliable initial sonographic triage (36).

### Innovation and domain robustness

Unlike previous studies where AI analyzed directly acquired ultrasound images, our study applied an AI to interpret images transmitted via a teleultrasound platform. The images were collected from 19 community institutions using various ultrasound devices, thus constituting a natural multicenter validation set with inherent domain diversity. The commercial AI system under investigation was trained on large-scale multicenter data employing domain generalization techniques specifically designed to enhance robustness against domain shift (37). Its strong agreement with expert interpretations in this heterogeneous environment provides preliminary validation of its robustness against inter-device variability.

However, challenges remain, including potential artifacts from image compression and novel device types. To further enhance robustness, we propose implementing a quality-aware inference pipeline that would assess input images for excessive compression, blurring, or signal deficiency prior to analysis. Low-quality images would be rejected with prompts for reacquisition. Additionally, incorporating uncertainty quantification would allow the system to flag low-confidence predictions for necessary human expert review, thereby establishing a safety net.

### Limitations and future directions

This study has certain limitations. First, the sample size of thyroid nodules classified as C-TIRADS 4A or higher in the study was limited, with no C-TIRADS 5 nodules included. The representation of rare nodule subtypes was insufficient to fully assess the accuracy of AI-assisted teleultrasound for these cases. However, our study provides preliminary evidence for the most common and diagnostically challenging suspicion nodules (C-TIRADS 4A-4C), which are frequently encountered in community health institutions.

Second, the use of expert consensus instead of pathological results as the reference standard introduces potential diagnostic bias. The study was limited by the difficulties in patient referral from community healthcare settings and suboptimal patient compliance. However, the findings hold significant value for primary care institutions lacking access to pathological diagnostics. The study not only provides primary care physicians with a reliable decision-support tool but also offers evidence supporting the broader and deeper application of AI technology in primary care settings.

Third, since only nodules classified as ≥C-TIRADS 4A were included in the AI system analysis, our study is subject to spectrum bias, which may lead to an overestimation of diagnostic performance. Nevertheless, this design was intentional to focus on the clinical scenario of triaging indeterminate nodules—precisely the context in which AI assistance could have the greatest impact in primary care.

Finally, although the inclusion of 19 heterogeneous institutions introduces variability, this multicenter diversity simultaneously strengthens the generalizability and external validity of our findings.

Future research should address these limitations through prospective, multicenter trials with preregistered analysis plans, employing histopathology as the gold standard. The primary objective should be to determine whether AI-assisted teleultrasound can facilitate implementation of the 2025 ATA Guidelines for Differentiated Thyroid Cancer (38), particularly by providing the consistent, longitudinal data necessary to support dynamic risk stratification and tailored long-term surveillance. Additionally, integrating multimodal diagnostic approaches with AI-assisted teleultrasound could improve the diagnostic accuracy. Subsequent studies should also implement calibration and decision-curve analyses to evaluate the clinical utility of AI when continuous risk scores are available.

## Conclusion

This study demonstrates that AI-assisted teleultrasound can substantially enhance the diagnostic agreement of community medical institutions for thyroid nodules of C-TIRADS 4A or higher, achieving assessment consistency comparable with expert teleultrasound reviewers. The AI system enables accurate nodule classification, reducing unnecessary referrals and improving workflow efficiency. More importantly, by providing reliable initial triage, it holds significant potential to optimize the downstream clinical pathway, including the selection of nodules for FNA and guidance in the management of indeterminate cytology—an area of critical importance and ongoing debate in thyroidology. This study provides a foundation for the broader application of AI into teleultrasound workflows for diagnostic support and quality control, ultimately supporting early detection and active surveillance of thyroid cancer.

## Data Availability

The original contributions presented in the study are included in the article/supplementary material. Further inquiries can be directed to the corresponding authors.
